# Surgical Management of Infective Endocarditis Complicated by Budd-Chiari Syndrome

**DOI:** 10.7759/cureus.70979

**Published:** 2024-10-07

**Authors:** Daiki Hirayama, Susumu Manabe, Norihisa Yuge

**Affiliations:** 1 Cardiac Surgery, International University of Health and Welfare, Narita Hospital, Chiba, JPN

**Keywords:** budd-chiari syndrome, cardiopulmonary bypass, infective endocarditis, mitral valve replacement, protein-c deficiency

## Abstract

Budd-Chiari syndrome (BCS) is a relatively rare comorbidity, particularly in patients undergoing cardiac surgery. The difficulty arises when we try to drain blood from the obstructed lower body circulation to establish extracorporeal circulation. Herein, we describe a patient who developed a persistent fever after undergoing neurosurgery for a head arteriovenous fistula, wherein blood cultures confirmed *Staphylococcus aureus* infection. The patient exhibited hyperbilirubinemia, hyperammonemia, and transient loss of consciousness. Transthoracic echocardiography showed moderate mitral regurgitation and 3 cm vegetation on the mitral valve. Imaging identified a thrombus in the right hepatic vein, stenosis of both the inferior vena cava and left hepatic vein, and esophagogastric and splenic varices. These findings led to establishing a diagnosis of infective endocarditis and BCS. The patient subsequently underwent mitral valve replacement with a mechanical valve. There are only a few case reports describing successful cardiac surgery in patients with BCS. Hence, we would like to report our surgical treatment of infective endocarditis complicated with BCS.

## Introduction

The clinical characteristics of patients with Budd-Chiari syndrome (BCS) are highly variable and often nonspecific. Patients commonly present without any clinical symptoms. The combination of abdominal pain, fever, hepatomegaly, and newly developed ascites, which are typically considered indicative of BCS, is relatively rare. Therefore, screening for hepatic venous outflow tract obstruction in any patient presenting with acute or persistent liver abnormalities is crucial. BCS is a rare condition characterized by the obstruction or stenosis of the main hepatic vein or hepatic inferior vena cava (IVC), leading to portal hypertension [1]. The increase in portal pressure results in the formation of collateral circulation.

There are limited case reports documenting cardiac surgery in patients with BCS [2,3]. Extracorporeal circulation in patients with BCS is considered difficult due to insufficient blood drainage from the obstructed lower body circulation. Only a few case reports have described successful cardiac surgery outcomes in patients with BCS. We aim to report our surgical treatment for a patient with infective endocarditis complicated with BCS.

## Case presentation

**Figure 1:**
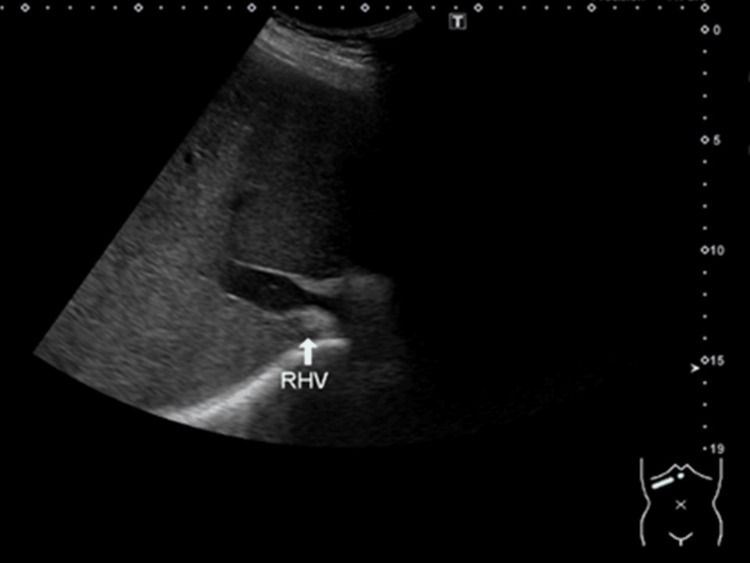
The abdominal ultrasound image demonstrates stenosis of the right hepatic vein (RHV), indicated by the arrow.

**Figure 2:**
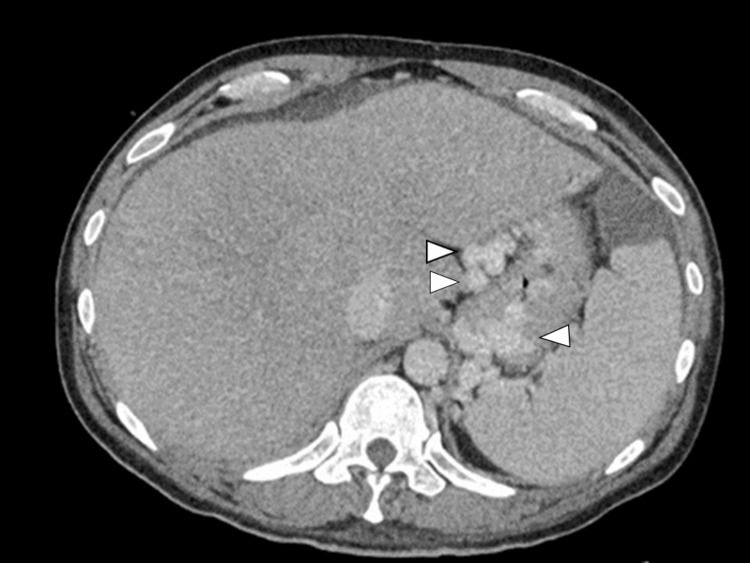
The contrast-enhanced CT scan indicates esophagogastric varices, marked by the arrowhead.

**Figure 3:**
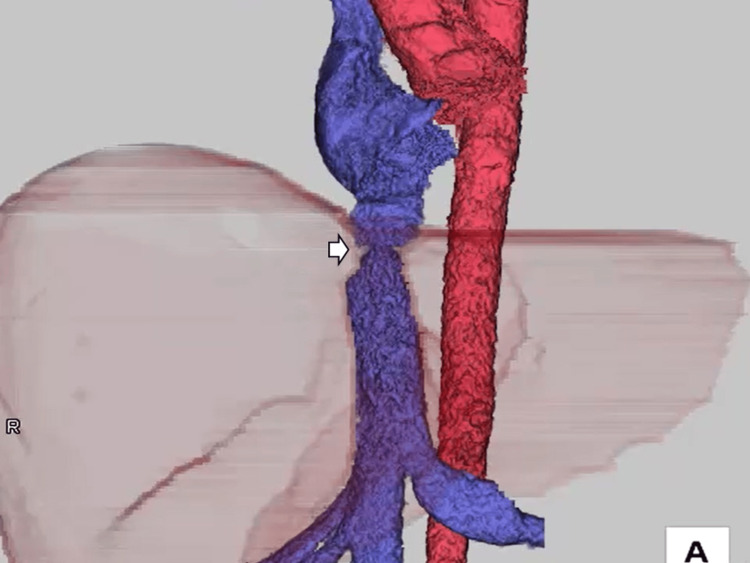
The contrast-enhanced CT scan indicates stenosis of the inferior vena cava (IVC), indicated by the arrow.

A 46-year-old man had sustained a fever after neurosurgical treatment for a head arteriovenous fistula. Blood culture was positive for *Staphylococcus aureus*. Transthoracic echocardiography revealed moderate mitral valve regurgitation with large mobile vegetation (3 cm). Simultaneously, he also had liver failure (aspartate transaminase: 1456 U/L, alanine transaminase: 250 U/L) and hyperammonemia (136 μg/dL), which resulted in a transient loss of consciousness. Abdominal ultrasound revealed hepatomegaly and a partial thrombus in the right hepatic vein (Figure 1). A thrombus was identified in the hepatic vein, prompting the initiation of heparin therapy. The contrast-enhanced computed tomography (CT) revealed stenosis of the IVC and left hepatic vein (Figure 2) with esophagogastric and splenic varices (Figure 3).

Eventually, he was diagnosed with infective endocarditis complicated by BCS. We planned surgical treatment for infective endocarditis, without transesophageal echocardiography (TEE) because of the risk of rupture of esophageal varices. Our plan involved establishing extracorporeal circulation by utilizing venous drainage from both the superior vena cava (SVC) and the femoral vein due to suspected IVC stenosis. Temperature management during cardiopulmonary bypass was set at 32°C; the total flow was 2.5-3.0 L/min/m^2^. Cannula sizes of 26 Fr, 32 Fr, and 29 Fr for the SVC, IVC, and femoral vein, respectively, were prepared. If these sources did not provide adequate flow, we would consider supplementing with additional drainage from the IVC. The venous drainage system for cardiopulmonary bypass was configured with three separate branches.

**Figure 4:**
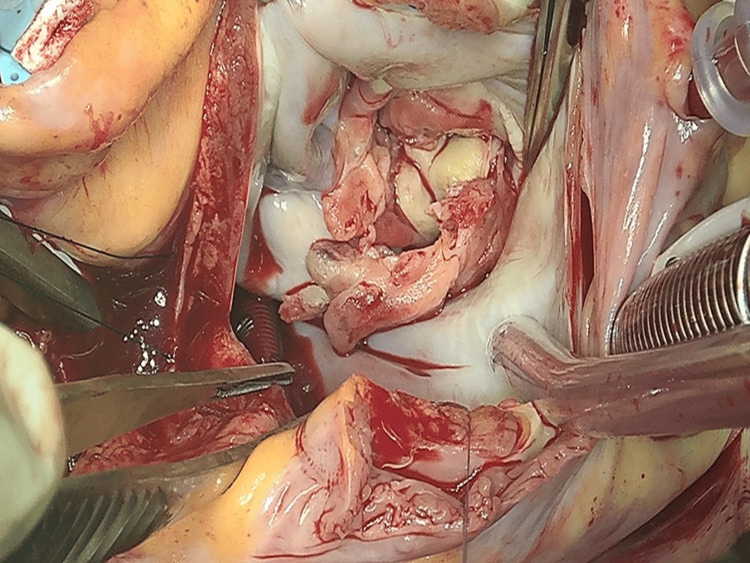
The intraoperative photograph showing the destruction of both leaflets.

A median sternotomy was performed, and extracorporeal circulation was established by bicaval cannulation. The SVC was cannulated from the right atrium (RA) and IVC from the femoral vein. However, we could not attain a sufficient extracorporeal blood flow probably due to the insufficient blood drainage from the IVC (50% of the total flow). Then, we added extra cannulation from RA into the IVC and attained sufficient extracorporeal blood flow. Cardiac arrest was induced with antegrade cardioplegia after cross-clamping the aorta. The mitral valve was exposed via the superior trans-septal approach. Valve analysis revealed huge vegetation affecting both leaflets and mitral annulus (Figure 4). The mitral valve was totally resected and replaced with a 29-mm mechanical valve. Extracorporeal circulation was weaned off uneventfully, and the patient was transferred to the ICU in stable condition. The total extracorporeal circulation time was 115 min. The patient was extubated on the day of surgery and was discharged without any postoperative complications. Blood tests revealed decreased protein C activity and gene analysis confirmed the diagnosis of protein C deficiency. Postoperatively, warfarin therapy was initiated; the patient has continued its use to the present time.

## Discussion

BCS is a rare disease characterized by portal hypertension due to occlusion or stenosis of the hepatic vein or IVC. The etiology of BCS is typically classified into primary and secondary forms. Primary BCS is attributed to thrombosis, vascular malformation, coagulation abnormalities, or myelodysplastic syndromes. Particularly, hypercoagulable status, such as protein C or S deficiency, is closely related to BCS [4]. In this case, hepatic dysfunction was identified after confirming the infection, leading to the diagnosis of BCS. Our case also showed protein C deficiency. Secondary BCS typically results from external blockages caused by factors such as tumor compression or invasion. Notably, several studies have reported cardiac surgery as a contributing factor to secondary BCS [5,6]. The safety of extracorporeal circulation in patients with BCS has not been established. Ishikawa and Sakamoto reported the successful establishment of extracorporeal circulation by cannulating the SVC and collateral circulation via the IVC with TEE [2].

**Figure 5:**
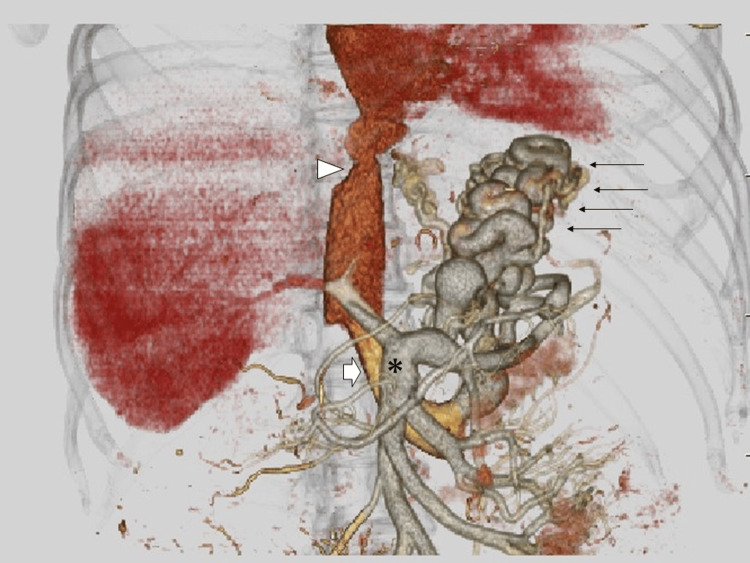
Postoperative contrast-enhanced CT A catheter was placed in the superior mesenteric artery (SMA), followed by injection of a contrast agent and subsequent CT imaging. The scan shows inferior vena cava (IVC) stenosis (▷), renal vein (⇨), gastrorenal shunt (→), and superior mesenteric vein (＊).

We planned to establish extracorporeal circulation using venous drainage from both the SVC and the femoral vein. If this approach did not provide sufficient flow, additional drainage from the IVC would be considered. The venous drainage setup for cardiopulmonary bypass was arranged in three separate segments. The reasons for this plan are as follows: in this case, the patient had esophageal varices, and considering the risk of rupture, the insertion of TEE was impossible. Because we could not confirm the position of the venous cannulation using TEE, there was concern about the potential misplacement into collateral circulation when inserting the venous cannula into the IVC. The initial attempt to cannulate the SVC via RA and IVC via the femoral vein was unsuccessful, probably due to multiple stenosis. Particularly, one IVC stenosis remained after the drainage of collateral circulation into the IVC (Figure 5).

In cardiac surgery complicated by BCS, proper cardiopulmonary bypass planning is of utmost importance. (1) If TEE insertion is feasible, venous cannulation should be performed in the SVC under TEE guidance to establish extracorporeal circulation. (2) If TEE insertion is not feasible, venous cannulation should be performed via the SVC and femoral vein to establish extracorporeal circulation. If sufficient blood flow cannot be obtained, additional venous drainage from the IVC should be considered.

Therefore, for the safe establishment of extracorporeal circulation in patients with BCS, it is mandatory to plan an individual approach in accordance with the variation of venous stenosis.

## Conclusions

This case underscores the complexities of managing infective endocarditis complicated by BCS, emphasizing the importance of tailored surgical strategies to prevent complications and ensure patient stability.
